# The Influence of the Teacher’s Prosocial Skills on the Mindwandering, Creative Intelligence, Emotions, and Academic Performance of Secondary Students in the Area of Physical Education Classes

**DOI:** 10.3390/ijerph17041437

**Published:** 2020-02-24

**Authors:** Ruben Trigueros, Marta García-Tascón, Ana M. Gallardo, Antonio Alías, José M. Aguilar-Parra

**Affiliations:** 1 Department of Psychology, Hum-878 Research Team, Health Research Centre, University of Almería, 04120 Almería, Spain; 2 Faculty of Sports Science, Univeridad Pablo de Olavide, 41704 Seville, Spain; margata@upo.es; 3 Faculty of Sports, Saint Anthony Catholic University, 30107 Guadalupe-Murcia, Spain; amgallardo@ucam.edu; 4 Department of Education, University of Almería, 04120 Almería, Spain; aag344@ual.es

**Keywords:** prosocial and antisocial behavior, mindwandering, intelligence creative, emotional state, physical education

## Abstract

Physical Education classes are a key context for the development of creativity due to interactions with peers and the resolution of complex motor skills. Therefore, the aim of this study is to analyze the influence of the teacher’s social behaviors on the mindwandering, emotional state, and academic performance of Physical Education students. The study involved 606 high school students and 36 physical education teachers. A structural equation model was used to analyze the relationship between the variables of the study. The results showed how the teacher’s prosocial and antisocial behaviors had a negative influence on mindwandering. In turn, mindwandering negatively predicted creative intelligence. Likewise, creative intelligence positively predicted a positive emotional state and academic performance and negatively predicted a negative emotional state. Finally, a positive emotional state positively predicted academic performance, while a negative emotional state predicted it negatively. Therefore, the results achieved in the study showed how mindwandering should be promoted in the educational field as a means of encouraging creativity and therefore increasing the well-being of students, which is conducive to academic performance.

## 1. Introduction

Current studies in the area of Physical Education (PE) have focused on the motivational and social processes that encourage students to participate in PE classes [1]. The main reason for this is that PE classes contribute to the promotion and consolidation of a series of adaptive habits related to personal health and well-being, through the learning of concepts, attitudes, and motor skills and abilities [2]. To this end, the teacher plays a decisive role, given his influence on the dynamics that are generated during PE classes and, at the same time, is in charge of managing the school curriculum [3]. In addition, with respect to learning motor skills and abilities, students must play an active role, since “trial and error” is required for learning, and creativity is a fundamental element, since it is a mechanism for effective problem management [2,4]. Therefore, in this study we propose to analyze the influence of teachers’ social behaviors on the mindwandering, creative intelligence, emotional state, and academic performance of students in secondary education.

### 1.1. Social Behaviours

The self-determination theory suggests that the behaviours that the teacher can have vary between two opposite dimensions: autonomy support versus control [3]. Autonomy support is related to prosocial behaviour, since it refers to establishing positive, empathic, cooperative, and socially responsible relationships in order to benefit their students [5]. On the other hand, control is related to antisocial behaviour, since it refers to the set of behaviours aimed at harming, damaging, or despising others, seeking only personal satisfaction [6]. Educational research has traditionally focused on antisocial behavior, showing that it is negatively related to motivation [7] and academic success [7], and that it is positively related to depression, anxiety, and stress [8,9]. In recent years, however, studies have focused on prosocial behaviors, which are closely related to the motivation to study [10], academic performance [10], and the satisfaction of psychological needs [11].

### 1.2. Mindwandering

Mindwandering refers to those mental processes in which attention moves from the current task to the internal thoughts generated by the self [12]. In this sense, mindwandering is one of the mental activities that is most often repeated, and which causes a person’s mind to move away from the task at hand, remaining in his or her internal thoughts as memories of the past or events that are about to happen. This happens in any of the activities of daily life, and there are studies that state that around 50% of the time adolescents spend in class they remain absorbed in their own thoughts [13].

Mindwandering fulfills two main functions: adaptive versus maladaptive. In this sense, it is associated with a wide variety of benefits, including future planning, problem solving, and creative thinking [14]. However, it has a negative effect, including poor performance on tasks, low morale, and discomfort [13,15]. In addition, mindwandering was consistently associated with poorer performance on tasks related to attention and comprehension during reading [16,17]. In this sense, a study by Unsworth and McMillan [18], related to the reading comprehension of high school students, showed that those students who were interrupted by questions while reading a text showed a negative effect on reading comprehension. This was due to the fact that tasks of a certain difficulty require a great mental effort for their execution, while simple tasks do not require so much effort. On the other hand, a study carried out by Marchetti, Koster, and De Raedt [19] showed that mindwandering negatively affected life satisfaction, causing the appearance of negative thoughts, which are related to negative emotions [20].

### 1.3. Intelligence Creativity

Although there are multiple definitions of the concept of intelligence and creativity, both definitions tend to share common frameworks. In this sense, intelligence is defined as the ability of an individual to adapt, change, or select an environment [21]. As for creativity, it is defined as the process of giving or creating something new and useful at the same time [22]. In this way, for an individual to adapt to multiple situations in the environment it is required for him to imagine, and to create a vision of how that environment should be and how it can become a reality. Therefore, creativity is a cognitive phenomenon that is found in all people, although not equally developed (see Garrett [23]). In this sense, the creative process can distinguish a series of factors [24]: cognitive, which refers to the capture and processing of information; affective, which refers to the elements that appear to be central to the mobilization of creative potential (e.g., motivation, emotion, self-esteem, etc.); and environmental, which refers to the conditions, terrain, or climate that facilitate the development and updating of creative potential.

The development of creativity in education allows freedom of thought and stimulating communication. If the classroom environment is attractive and generates ideas and resources, students will feel free to be, think, feel, and experiment in their own way, knowing in advance that they are accepted as they are and that their contribution will be valued [21,22]. Therefore, several studies in the educational field have shown that creativity has been positively related to openness to experiences and extraversion [25], academic performance [24], self-concept [26], and learning new skills and motor abilities [27]. In contrast, creativity has been negatively related to anxiety and academic stress [28], cognitive decline [29], and social isolation [30].

### 1.4. Emotional State

According to the Individual Zones of Optimal Functioning (IZOF) model developed by Hanin [31], emotion is a situational, multimodal, and dynamic manifestation, basic to human functioning. Therefore, emotions constitute an evaluative assessment of an external situation that produces both the psychological and physiological activation of the organism, and that determines our actions. This model presents five basic dimensions (form, content, intensity, time, and context) to describe the optimal and functional structure of the individual, as well as a functional explanation of the dynamics of the relationships between emotions and performance based on the detailed description of people’s subjective experiences. This is especially important in the educational context, as it is believed that emotions can have a certain influence on students’ self-regulation by favouring their motivation to learn, study, and strive.

Despite this, the studies that have existed up to now in the field of PE are very fragmented, and there are theories and studies that deal with emotions individually (e.g., shame [32]) or studies on the unique functions of emotions such as the Positive and Negative Affect Schedule (PANASN [33]). However, emotions tend to appear together, which means that several appear at the same time, with different studies omitting the effect that several emotions may have on academic achievement, performance, and, in short, the achievement of academic objectives [34]. Therefore, the present study aims to measure the emotions jointly depending on the valence.

### 1.5. Objective and Hypothesis

The hypothesis of this study is as follows: Prosocial and antisocial teacher behaviour will positively predict mindwandering; (2) mindwandering will positively predict creative intelligence; (3) creative intelligence will positively predict positive emotions and negatively predict negative emotions; and (4) positive emotions will positively predict academic performance, and negative emotions will negatively predict academic performance.

## 2. Method

### 2.1. Participants

The present study involved 323 boys and 283 girls, aged 15–18 years (M = 16.83; SD = 1.12; Table 1). In addition, 36 PE teachers, 22 male and 14 female, aged 31–54 years, participated (M = 42.65; SD = 5.48; Table 2). The participants belonged to several educational centers in a province of Almeria.

The sample used was incidental and not probabilistic, depending on the secondary schools and the students we had access to. The inclusion criterion for participation in the study was: to provide informed advice signed by parents or a legal guardian.

### 2.2. Measures

Prosocial and antisocial behaviours of PE teachers. The Prosocial and Antisocial Behavior in Sport Scale, (PABSS; Kavussanu and Boardley [35]) validated and adapted to the context of PE by Trigueros, Alias, Gallardo, García-Tascón, and Aguilar-Parra [36] was used. Teachers rated prosocial and antisocial behaviors toward their students during PE classes. The teachers completed the four item scale to evaluate the prosocial behavior factor and the four item scale to evaluate the antisocial behavior factor; a fifth item was eliminated from the latter factor referring to “insulting my students”, since this behavior never occurs in a PE classroom. For our study, we modified the word “teammate” from the original scale to the word “students”. The response scale ranges from 1 (Strongly Disagree) to 7 (Strongly Agree).

Mind-Wandering. The Mind-Wandering Questionnaire (MWQ; [17]) was used, validated, and adapted to the Spanish context of PE by Trigueros, Aguilar-Parra, Cangas, and Álvarez [37]. The scale was preceded by the heading “In my PE classes…” and consists of five items (e.g., I do the exercises without paying attention) with only one factor. The students had to answer according to a Likert scale ranging from 1 (almost never) to 6 (almost always).

Creative Intelligence. The CREA test was used, validated by Corbalán, Martínez, Alonso, Donolo, Tejerina, and Limiñana [38]. The purpose of the test is the appreciation of creative intelligence through a cognitive evaluation of individual creativity according to the indicator of question generation, in the theoretical context of search and problem solving. The test consists of three sheets from which the subject has to generate all kinds of questions suggested by the drawings. The keynote of the test is: You will be presented with an illustration. Your task is to write briefly all the questions you can ask about what the picture represents. Try to ask as many questions as possible in four minutes. For a more detailed explanation of your assessment, see Corbalán et al. [38]. The psychometric properties of the instrument have been demonstrated in multiple investigations in different geographical contexts [39,40].

Emotional State. The Emotions in Physical Education Questionnaire validated by Trigueros, Aguilar-Parra, Cangas, and Álvarez was used [34]. The questionnaire is headed by the following sentence “During PE classes…”, making up a total of 32 items distributed among the eight factors that make up the scale (e.g., anxiety, shame, boredom, hopelessness, fun, pride, tranquility, and confidence). The students had to answer by means of a Likert scale from 1 (totally disagree) to 7 (totally agree).

Academic Performance. To measure academic performance, the grades obtained at the end of the academic year were taken into account. The grades have been distributed as follows: 1 (failing), 2 (passing), 3 (good), 4 (notable), and 5 (outstanding).

### 2.3. Procedure

In order to carry out the study, permission was requested from the educational centres and the parents of the participants, as they were minors. Previously, it was explained to the teachers that the collection of the data occurred before the beginning of the classes and respecting the anonymity of the students’ answers. Later, it was explained to the teachers and students that they were going to participate in a study on their academic performance and experiences related to the PE classes. The questionnaires were administered at the end of the 2018/2019 academic year, specifically at the beginning of the PE classes.

The administration of the questionnaires was carried out under the supervision of the principal investigator, with more than 11 years of experience in the field of research, who explained and resolved the doubts that arose when completing the questionnaire. The application of the questionnaires was carried out in the schools. The estimated time to complete the questionnaire was around 20 min.

This study was carried out in accordance with the Declaration of Helsinki. Ethics approval was obtained from the Research Ethics Committee of the University of Almeria, Spain (Ref. UALBIO 2019/014).

### 2.4. Data Analysis

First, descriptive statistical analyses, bivariate correlations, and reliability analyses were performed using the SPSS v.25 statistical program. Then, a structural equation model was made using AMOS v.20 program in order to test the relationships established in the hypothesized model.

**Figure 1:**
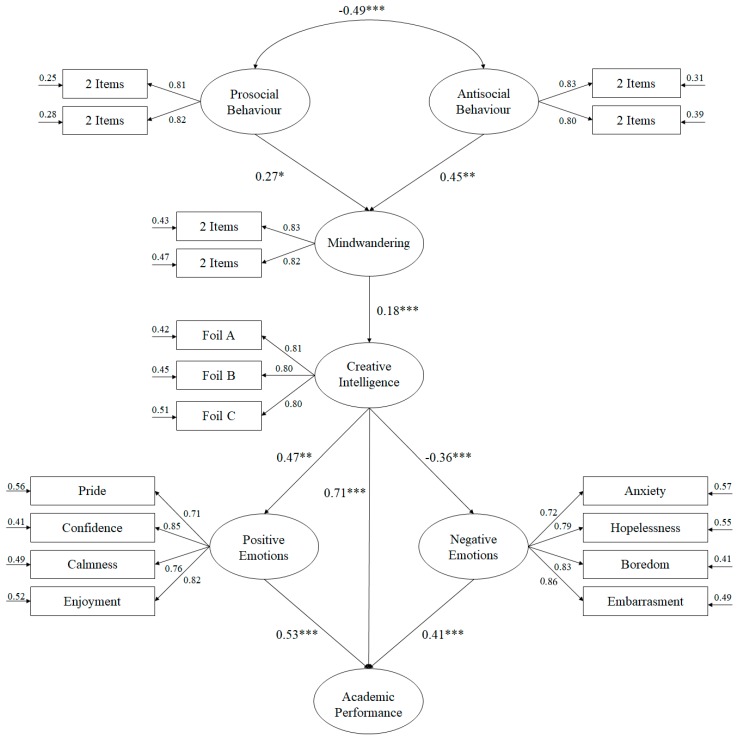
Structural equation model showing the relationships between the various factors that make up the study. All parameters are standardized and statistically significant. Note: *** *p* < 0.001; ** *p* < 0.01; * *p* < 0.05.

In the Structural Equation Model (see Figure 1), the maximum likelihood estimation method was used, along with the bootstrapping procedure. In addition, a set of adjustment indexes was taken into consideration to accept or reject the model tested [41]: the chi-square coefficient divided by degrees of freedom (*χ2*/*df*), with values below 3 considered acceptable; the CFI (Comparative Fit Index), IFI (Incremental Fit Index), and TLI (Tucker Lewis Index) incremental indexes that show a good fit with values of 0.95 or higher; RMSEA (Root Mean Square Error of Approximation) plus its confidence interval (CI) at 90% and SRMR (Standardized Root Mean Square Residual), which are considered acceptable with values equal to or less than 0.06 and 0.08 respectively. However, these fit rates should be interpreted with caution as they are too restrictive and difficult to achieve when testing complex models [42].

## 3. Results

### 3.1. Preliminary Analysis

The descriptive statistics, bivariate correlations, and reliability analysis through Cronbach’s α between the study variables can be seen in Table 3. Cronbach’s α scores were above 0.70.

The correlation analyses reflected positive associations, all of which were significant, between each of the study factors, except those related to negative emotions.

### 3.2. Structural Equation Modeling

Before starting the analyses of the hypothesized model and studying the relationships between the variables belonging to the model, a reduction of the number of latent variables was previously carried out. This is especially appropriate when the complexity of the model requires it (see McDonald and Ho [43]). Specifically, the latent variables used were: creative intelligence included three indicators (Foil A, Foil B, and Foil C [38]); positive emotions included four factors (fun, pride, tranquility, and confidence [36]); and negative emotions included four indicators (boredom, hopelessness, anxiety, and embarrassment [36]). Similarly, for prosocial and antisocial behaviours it was necessary to separate the four items measuring each of these latent factors into two indicators for each. Finally, for mindwandering, we needed to separate the five items that measure it into two indicators.

The adjustment rates of the hypothesized model were as follows: χ^2^ (244, N = 606) = 847.36, χ^2^/df = 3.31, *p* < 0.001, IFI = 0.097, TLI = 0.97, CFI = 0.97, RMSEA = 0.059. (IC 90% = 0.053–0.062), SRMR = 0.038. These results fit the established parameters, so we can accept the proposed model as adequate [41].

The correlations obtained between the different factors that make up the model are described below (Figure 1):

(a) The prosocial behavior positively predicted the mindwandering (*β* = 0.27, *p* < 0.05). Similarly, antisocial behavior positively predicted mindwandering (*β* = 0.45, *p* < 0.01).

(b) Mindwandering positively predicted creative intelligence (*β* = 0.18, *p* < 0.001).

(c) Creative intelligence positively predicted positive emotions (*β* = 0.47, *p* < 0.001) and academic performance (*β* = 0.71, *p* < 0.001); in contrast, it negatively predicted negative emotions (*β* = −0.36, *p* < 0.001).

(d) Positive emotions positively predicted academic performance (*β* = 0.53, *p* < 0.001), while negative emotions negatively predicted academic performance (*β* = 0.41, *p* < 0.001).

## 4. Discussion

The present study has analyzed, in the context of PE, the influence of the teacher’s prosocial skills on mindwandering, creative intelligence, emotions, and academic performance.

The results revealed that the teacher’s antisocial behaviors were positively related to mindwandering, while the teacher’s prosocial behaviors were negatively related to mindwandering. However, the results obtained in the present study cannot be compared with similar studies in the field of PE classes, although they can be compared with previous studies in the field of social psychology. In this sense, a study conducted by Roussou [44] with adults showed how prosocial behaviors are negatively related to mindwandering. Similarly, several previous studies in the field of mental health have shown how antisocial behaviors have been positively related to mindwandering [45,46]. The results between teacher social behaviors and student mindwandering could be explained in such a way that mindwandering is a phenomenon inherent to all human beings, our attention fluctuating continuously between our internal and external world, occurring during any activity [47], including during PE classes, a context in which attention is required to be maintained for longer periods of time and causing greater attention to be spent.

The results have shown how mindwandering posthumously predicted creative intelligence. As with the previous result, this relationship cannot be compared with previous studies in the field of PE classes. However, in the educational field, a study by Ellamil, Dobson, Beeman, and Christoff [48] with art students showed that mindwandering favoured divergent thinking, creativity, and artistic work. Similarly, a study conducted by Leszczynski, Chaieb, Reber, Derner, Axmacher, and Fell [49] with adults showed that mindwandering was positively related to creative problem solving and the planning of daily routine tasks. These results can be explained by the fact that mindwandering can enhance creative problem solving through the process of creative association of ideas, concepts, and/or representations, because several moments of internal reflection allow the best solution to be reached accidentally since the mental rumination of a problem leads to blockage [50,51].

Furthermore, the results have shown how emotional intelligence positively predicted positive emotions and academic performance, and negatively predicted negative emotions. These results have been shown to be similar to several previous studies in the field of PE classes. In this sense, a study conducted by Justo [52] with early childhood education students showed how creativity helped the learning and strengthening of students’ motor skills and abilities, thus favoring their academic performance. Similarly, a study conducted by Spendlove [53] with fine arts students during high school showed that those students with high levels of creativity favored their performance, learning, and academic achievement. On the other hand, a study conducted by Li and Yu [54] with high school students showed how creative intelligence was positively related to positive feelings such as self-efficacy, certain positive emotions (e.g., fun, pride, etc.), and academic performance. In addition, a study by Araya and Jimenez, [55] in the area of PE classes showed how high levels of creativity positively predicted positive emotions and negatively predicted negative ones. On the other hand, a study by Ivcevic, Brackett, and Mayer [56] showed that creativity correlated positively with emotional intelligence, the latter being a precursor of positive moods and a protector against negative emotions [57]. These results can be explained by the fact that PE students with higher levels of creativity allow students to solve motor problems more quickly and effectively, thus experiencing fun and joy, positive emotions. Conversely, students with low levels of creativity take longer to solve motor problems, triggering negative emotions such as frustration, hopelessness, boredom, or anxiety.

Finally, positive emotions positively predicted academic performance, while negative emotions predicted it negatively. These results have been shown to be similar to several previous studies in the educational field, although not in the field of PE classes. A study by Pekrun, Elliot, and Maier [58] showed that students with a positive emotional perception of the classroom showed greater interest and performance, which in turn led to higher academic achievement. Similarly, a study by Putwain, Sander, and Larkin [59] showed how the positive experiences experienced by students during classes favored the emergence of positive feelings, which had a positive influence on learning and academic performance. Therefore, the results of this study show how positive emotions are precursors of academic performance. In this sense, greater emotional satisfaction leads to a greater sense of emotional well-being, which in turn leads to greater involvement, attention, and motivation towards PE classes.

Despite the findings of this study, there are a number of limitations that should be taken into consideration. The first is that the collection of information has been based on self-administered questionnaires. The second limitation is that the findings of this study can be interpreted in many different ways, depending on the reader’s point of view. Finally, the present study has only taken into account the social behaviours of teachers, so future studies should provide evidence of the social behaviours of classmates and parents.

## 5. Conclusions

These results highlight the central role of mindwandering as a precursor to the creative intelligence of PE students. The study shows that student mindwandering cannot be reduced, regardless of the teacher’s role. Furthermore, from this study there is evidence that mindwandering is a precursor to creative intelligence and, therefore, to effective problem solving and higher academic performance. In this sense, teachers must generate strategies to try to reduce those intrusive episodes of neglect that interfere with learning, while at the same time they must try to open instances where students can actively direct their attention internally and take advantage of the potential of mental digression in the generation of creative thinking.

## Figures and Tables

**Table 1:** Description of the age of the participants (Students).

**Gender**	15yr	16yr	17yr	18yr
**Male**	81	68	91	83
**Female**	75	62	78	68

**Table 2:** Description of the age of the participants (Teachers).

**Gender**	31–38yr	39–46yr	47–54yr
**Male**	7	5	10
**Female**	4	6	4

**Table 3:** Descriptive statistics and reliability analysis.

Factors	*M*	*SD*	α	1	2	3	4	5	6	7
1. Prosocial	3.05	1.43	0.86		−0.34 **	0.19 ***	0.35 **	0.56 ***	-0.67 ***	0.52 ***
2. Antisocial	1.86	1.03	0.84			0.31 **	−0.02 *	−0.29 **	0.70 **	−0.78 **
3. Mindwandering	2.12	1.78	0.80				0.27 **	0.58 **	0.42 **	−0.12 **
4. Creative Intelligence	12.91	8.31	0.83					0.27 **	−0.14 **	0.84 **
5. Positive Emotions	5.25	1.54	0.81						−0.44 ***	0.48 ***
6. Negative Emotions	2.37	0.82	0.79							−0.35 ***
7. Academic Performance	3.12	0.68	0.78							

Note: *** *p* < 0.001; ** *p* < 0.01; * *p* < 0.05.
